# Changes in hypoxia level of CT26 tumors during various stages of development and comparing different methods of hypoxia determination

**DOI:** 10.1371/journal.pone.0206706

**Published:** 2018-11-09

**Authors:** Łukasz Kiraga, Łukasz Cheda, Bartłomiej Taciak, Kamila Różańska, Katarzyna Tonecka, Aleksandra Szulc, Krzysztof Kilian, Emilia Górka, Zbigniew Rogulski, Tomasz P. Rygiel, Magdalena Król

**Affiliations:** 1 Department of Physiological Sciences, Faculty of Veterinary Medicine, Warsaw University of Life Sciences, Warsaw, Poland; 2 Biological and Chemical Research Centre, Faculty of Chemistry, University of Warsaw, Warsaw, Poland; 3 Department of Immunology, Centre for Biostructure Research, Medical University of Warsaw, Warsaw, Poland; 4 Heavy Ion Laboratory, University of Warsaw, Warsaw, Poland; University of South Alabama Mitchell Cancer Institute, UNITED STATES

## Abstract

The aim of this study was to evaluate hypoxia level at various tumor developmental stages and to compare various methods of hypoxia evaluation in pre-clinical CT26 tumor model.

Using three methods of hypoxia determination, we evaluated hypoxia levels during CT26 tumor development in BALB/c mice from day 4 till day 19, in 2–3 days intervals. Molecular method was based on the analysis of selected genes expression related to hypoxia (*HIF1A*, *ANGPTL4*, *TGFB1*, *VEGFA*, *ERBB3*, *CA9*) or specific for inflammation in hypoxic sites (*CCL2*, *CCL5*) at various time points after CT26 cancer cells inoculation. Imaging methods of hypoxia evaluation included: positron-emission tomography (PET) imaging using [^18^F]fluoromisonidazole ([^18^F]FMISO) and a fluorescence microscope imaging of pimonidazole (PIMO)-positive tumor areas at various time points.

Our results showed that tumor hypoxia at molecular level was relatively high at early stage of tumor development as reflected by initially high *HIF1A* and *VEGFA* expression levels and their subsequent decrease. However, imaging methods (both PET and fluorescence microscopy) showed that hypoxia increased till day 14 of tumor development. Additionally, necrotic regions dominated the tumor tissue at later stages of development, decreasing the number of hypoxic areas and completely eliminating normoxic regions (observed by PET).

These results showed that molecular methods of hypoxia determination are more sensitive to show changes undergoing at cellular level, however in order to measure and visualize hypoxia in the whole organ, especially at later stages of tumor development, PET is the preferred tool. Furthermore we concluded, that during development of tumor, two peaks of hypoxia occur.

## Introduction

Cells become hypoxic when the oxygen demand exceeds the oxygen supply. Hypoxia arises in solid tumors due to various factors. The most important is the rate of cancer cells proliferation. Tumor growth depends upon the host tissue-derived blood vessels; however, these blood vessels normally support only selected parts of the tumor tissue and for a relatively short time. Tumor blood vessels are distinct from that in normal tissue and tumor blood supply is compromised as those vessels are often elongated, dilated and twisted [1]. Low oxygen levels inside the tumor, or sometimes also lactic acid synthesized as by-product of anaerobic glycolysis of tumor cells, stimulate expression of hypoxia-inducible factor 1α (*HIF1A*) transcription factor, which induce vascular endothelial growth factor (*VEGFA*) production, which in turn gives rise to tumor-derived angiogenesis [1,2].

The location, extent and relative number of hypoxic regions vary at different stages of tumor developmental. Oxygen deficiency initially develops in distant sites from blood vessels [3]. Solid tumors have characteristic pattern of growth, where proliferating cells are situated close to a functional blood vessel and necrotic cells are situated peripherally. The cells located between those regions are not sufficiently supplied by oxygen and they are considered to form hypoxic layer of the tumor [4]. Additionally, prolonged hypoxia of the tumor tissue leads to necrosis of the inner layer of the tumor mass [5].

Incorporation of the hypoxia-extend information in clinical tumor assessment is important for the development of personalized, hypoxia-based therapies [6]. Recent clinical trial data demonstrated how patients stratified by their hypoxic tumor status benefited from hypoxia-modified therapies compared with standard therapies. Therefore, there is an urgent need to improve patient stratification by determining the extent of hypoxia. The hypoxia assessment may aid not only radiation oncologists and surgeons, but also biotechnology and pharmaceutical companies in developing tumor hypoxia therapies or other new treatment strategies for hypoxic tumors. Additionally, evaluation of tumor hypoxia at various stages of development is also important from the scientific point of view in order to better characterize the studied models and understand processes that undergo inside the tumor mass [6].

## Materials and methods

### Cell culture and animals

Mouse colorectal carcinoma cell line CT26 was purchased from ATCC (Manassas, Virginia). Cells were cultured under optimal conditions: RPMI-1640 supplemented with 10% (v/v) heat-inactivated fetal bovine serum (FBS) and penicillin-streptomycin (50 U/mL) in standard culture conditions in an atmosphere of 5% CO_2_ and 95% humidified air at 37°C. The cell culture maintained among whole experiment was regularly *Mycoplasma* tested using PCR and agarose gel electrophoresis. Cell culture reagents were purchased from Sigma Aldrich (Saint Louis, Missouri).

For the experiments, BALB/c mice (females, 8–12 weeks old) purchased from the Center for Experimental Medicine of the Medical University of Bialystok, were housed in the conventional, non-SPF facility of the Medical University of Warsaw. At the day of inoculation, CT26 cells were harvested with 10% trypsin, washed and suspended in 1:1 mixture of ion-free PBS (Biowest, Nuaillé, France) and Matrigel Growth Factor Reduced (Corning Life Sciences, Corning, New York). Before the procedure, mice were anesthetized with combination of ketamine (75mg/kg Ketamine, Biowet, Pulawy, Poland) and xylazine (5mg/kg, Xylapan, Gorzow Wlk., Poland) injected intraperitoneally. Tumor cells (1.5 x 10^5^) suspended in 30ul of PBS and Matrigel mixture were inoculated intramuscularly in the right lower leg using 1ml insulin syringe (needle 27G). Euthanasia was performed by ketamine and xylazine mixture intraperitoneal overdose.

All experimental protocols were concordant with the national legislation and were approved by the First Local Ethical Committee in Warsaw. All procedures were performed under anesthesia, and all efforts were made to minimize suffering.

### Real-time qPCR

Gene expression was analyzed in tumor tissues obtained at 4, 7, 10, 14, 16, and 19 day after CT26 tumor inoculation. Tumors were homogenized and 20 mg of RNA-later stabilized tissue homogenate was used for RNA isolation on silica columns (A&A Biotechnology, Gdynia, Poland). One μg of total RNA was used for cDNA synthesis (Roche, Basel, Switzerland). Real-time qPCR was performed using a fluorogenic SYBR Green Master Mix kit (ThermoFisher, Waltham, Massachusetts) and a Mx3005P QPCR System (Agilent Technologies, Santa Clara, California). Data were analyzed using the comparative Ct method [7]. Sequences of key genes were obtained from PrimerBank [8]. Ten different genes were tested as possible housekeeping genes, indicated in supporting information file (S1 Table). Finally, results were normalized to eukaryotic elongation factor 2 (*EEF2*) housekeeping gene. In this study, we used 3 mice per group (time-point). This analysis has been performed in triplicate.

### PET-CT

Evaluation of hypoxia level in different time-points of tumor development was performed by intravenous administration of [^18^F]fluoromisonidazole ([^18^F]FMISO) followed by PET-CT imaging at the day 5, 7, 9, 12, 14, 16, and 18 after cancer cells inoculation. In each time-point 6–9 mice were analyzed. For each time-point of imaging a new [^18^F]FMISO synthesis was performed according to the previously optimized and described procedure [9]. Each mouse was placed in restrainer for tail vein injection and 10–20 MBq of [^18^F]FMISO (30–50 μl) was administered intravenously. Before PET-CT imaging, each mouse was anesthetized with 3–3,5% isoflurane (AERRANE, Baxter, Warsaw, Poland) delivered through a nose cone and placed on the heating pad. Mice were placed in the supine position in the scanner with the use of the animal bed. The 1,5–2% isoflurane anesthesia was maintained through entire image acquisition. The PET imaging, followed by CT, was performed using Albira PET/SPECT/CT Preclinical Imaging System (Bruker, Billerica, Massachusetts). Respiration was monitored with pressure pad connected to differential pressure transducers for low-range pressure monitoring—MP150 Acquisition System (Biopac, Goleta, California) during entire PET-CT examination. PET scans were performed 120 min after [^18^F]FMISO injection. Emission data were collected for 10 min. Spatial resolution of PET measurements was 1.1 mm. The scan parameters were set as follow: tube voltage was 45 kVp, tube current was 400 μA, and number of projections was 400. Minimal resolution of CT was 90 μm (CTbest). During whole experiment, each mouse was examined by PET-CT imaging 1 to 3 times (in different time-points of tumor development).

### PET analysis methods

Obtained PET and CT metadata were reconstructed and DICOM files of PET and CT images were fused using PMOD software, version 3.806, module PFUS (PMOD Technologies LLC, Zurich, Switzerland). Tumor shape on fused PET-CT image was contoured on each slide consisting part of tumor. Obtained VOI (volume of interest) was quantitatively analyzed using PBAS module of PMOD and data consisting kBq per ml of VOI was taken. Collected data was decay-corrected for T1/2 of ^18^F = 109,771 min related to time passed from radiotracer injection to the end of PET signal acquisition. Intensity of hypoxia in tumor tissue was evaluated by counting SUV (standardized uptake value), and %ID/ml (per cent of injected dose per ml of VOI). SUV value was calculated basing on commonly used formula [10]. Both SUV and %ID/ml values calculations were modified by the use of ID_corrected_ value instead of typically used ID (injected dose). Both formulas are specified in supporting information file (S1 Formulas). The ID_corrected_ reflects correctly injected sample and it is calculated by multiplying injected dose (after subtraction of radioactivity remaining in the syringe, decay corrected) by the ratio of radioactivity of the body without the tail to the whole body. We used VOIs of the whole body, the body without tail and collected total radioactivity [kBq] data of indicated VOIs using PBAS module. Correction of ID value allows calculations of desired values in more accurate way since it does not include radioactivity remaining in the tail connective tissue. Using ID_corrected_ value requires subtraction of tail weight (TW) from the whole-body weight (BW). Evaluation of volumes of hypoxic and non-hypoxic regions of tumors was performed using PBAS module. Hypoxic tissue was marked on PET image creating VOI of hot signal (above indicated threshold). The threshold was determined by measuring averaged signal from opposite tight muscle (not the one with the tumor). Volumes of created VOIs were measured in PBAS. Volume of non-hypoxic tissue was calculated by subtraction of volume of hypoxia from the volume of whole tumor, marked on the CT image. Depending on the tumor age of development non-hypoxic tissue may refer to normoxic tissue (until 12^th^ day) or necrotic tissue (from 14^th^ day). Surface rendering of different regions of 16-day tumor and whole mouse at the day 12 of tumor development was performed using P3D module of PMOD.

### Hypoxia examination using fluorescence microscopy

Hypoxia tissue regions were investigated by intravenous administration of pimonidazole (PIMO Hypoxyprobe-Green Kit) containing 100 mg pimonidazole HCl plus 1 unit of 4.3.11.3 mouse FITC-Mab (Hypoxyprobe, Burlington, Massachusetts) at the day 8, 11, and 14 after cancer cells inoculation. At each time-point 3 mice were analyzed. The PIMO solution 1.5 mg/mouse (100 μl) was intravenously injected 1–1.5 hours before the examination. PIMO is reductively activated in hypoxic cells. The activated intermediate forms stable covalent adducts with thiol (sulfhydryl) groups in proteins, peptides and amino acids [11,12]. The antibody reagent MAb1 binds to these adducts allowing their detection by immunochemical means. Perfusion regions were stained with Hoechst 33342 (ThermoFisher, Waltham, Massachusetts) administered intravenously 1 min before harvest (100 μl/mouse, concentration 9 mg/ml). Obtained tumors were frozen and cut with cryotome and sections from three tumor regions (periphery, centre and contralateral periphery—at least three sections from each tumor region); at least ten sections of each tumor were used for the analysis. To visualize hypoxic and perfused regions, a fluorescence microscope (Olympus BX60, Olympus, Tokyo, Japan) at 20× magnification was used. Threshold used for the analysis was set at 49 (for hypoxia) and 60 (for high hypoxia). The results are reflected as delta of fluorescence related to PIMO and the whole tumor fluorescence. The experiment was repeated three times. For hypoxia calculations, the MicroImage (Olympus, Tokyo, Japan) and ImageJ software have been used.

### Statistical analysis

**Fig 1:**
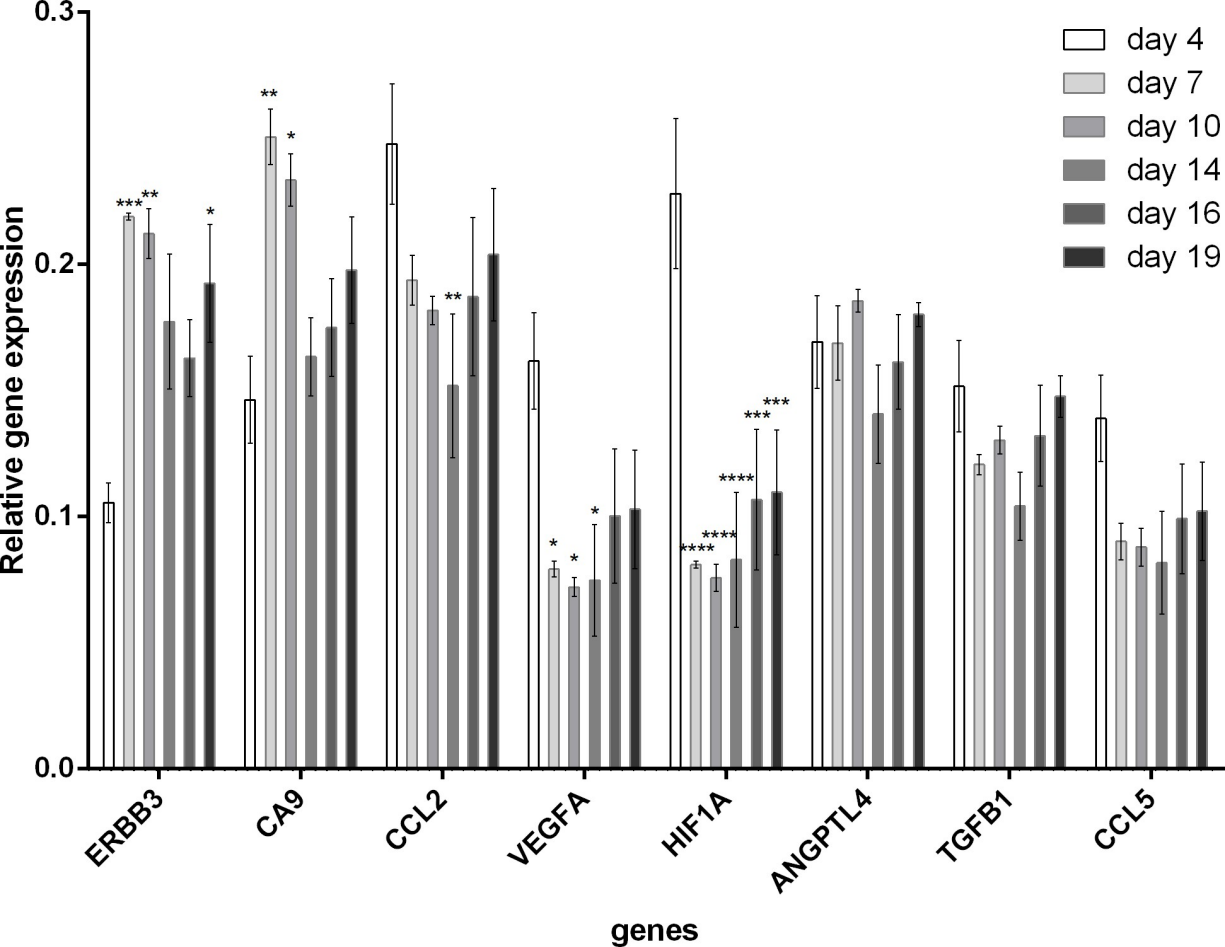
Expression analysis (real-time qPCR) of selected genes related to hypoxia at various stage of tumor development (from day 4 till 19 post CT26 cells inoculation).

The statistical analysis was conducted using Prism version 5.00 software (GraphPad Software, San Diego, California). The one-way ANOVA and Tukey HSD (Honestly Significant Difference) post-hoc test, Dunnett’s test were applied. The p-value <0.05(*) was regarded as significant whereas p-value <0.01(**) and p-value <0.001(***) as highly significant. In the graphs, error bars reflect standard error of the mean (SEM). Raw data set for the graphs shown in Figs 1, 3B, 3C, 4B and 5 can be found in supporting information file (S1 Dataset).

## Results

### Gene expression analysis

Analysis of the gene expression related to hypoxia (*HIF1A*, *ANGPTL4 –Angiopoietin-like 4*, *TGFB1 –Transforming Growth Factor Beta 1*, *VEGFA*, *ERBB3 –Erb-B2 Receptor Tyrosine Kinase 3*, *CA9 –Carbonic anhydrase 9*) or specific for inflammation in hypoxic sites (*CCL2 –C-C motif chemokine 2 precursor*, *CCL5 –C-C motif chemokine ligand 5*) at day 4, 7, 10, 14, 16, and 19 after CT26 cancer cells inoculation showed that the tumor hypoxia at molecular level was relatively high at the early stage of the tumor development (Fig 1). Particularly, the expression of high *HIF1A* and *VEGFA* was high at the early time-point and decreased in time. *HIF1A* and *VEGFA* expressions were significantly higher at the day 4 than at the other days (p<0.001 and p<0.01, respectively). Other hypoxia related genes (*CA9* and *ERBB3*) showed the highest expression at day 7 (both p<0.001) and day 10 (p<0.01 and p<0.001, respectively) compared with day 4 (Fig 1). Whereas, other analyzed genes did not show any significant changes in expression during tumor development.

### PET analysis

**Fig 2:**
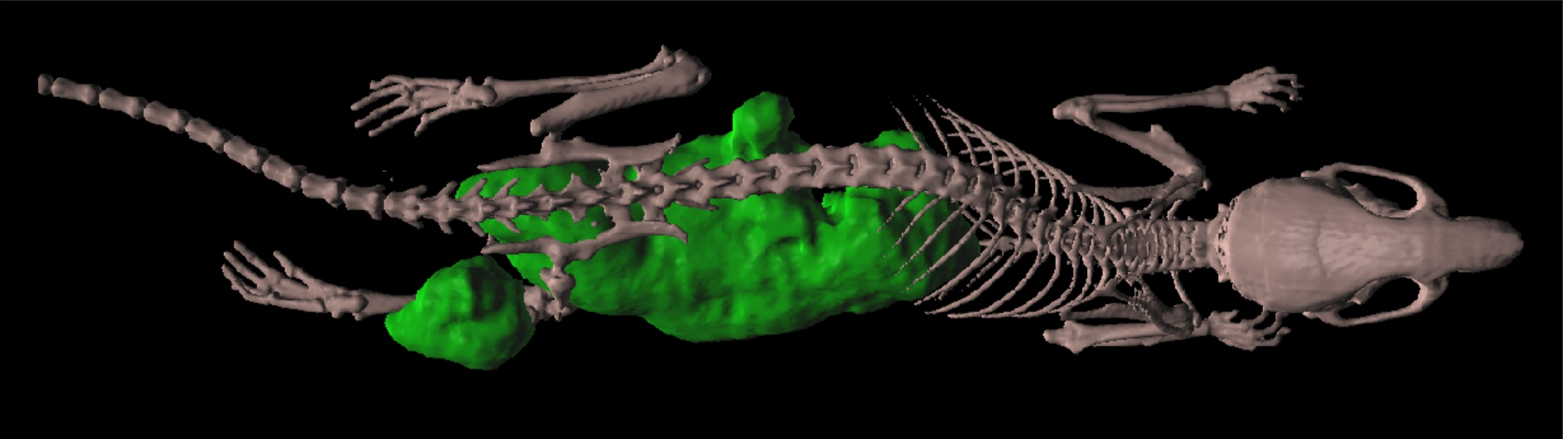
Mouse at the day 12 of tumor development after [^18^F]FMISO administration, 3D surface rendering of PET-CT image. Green color reflects hypoxic regions in the whole mouse body.

**Fig 3:**
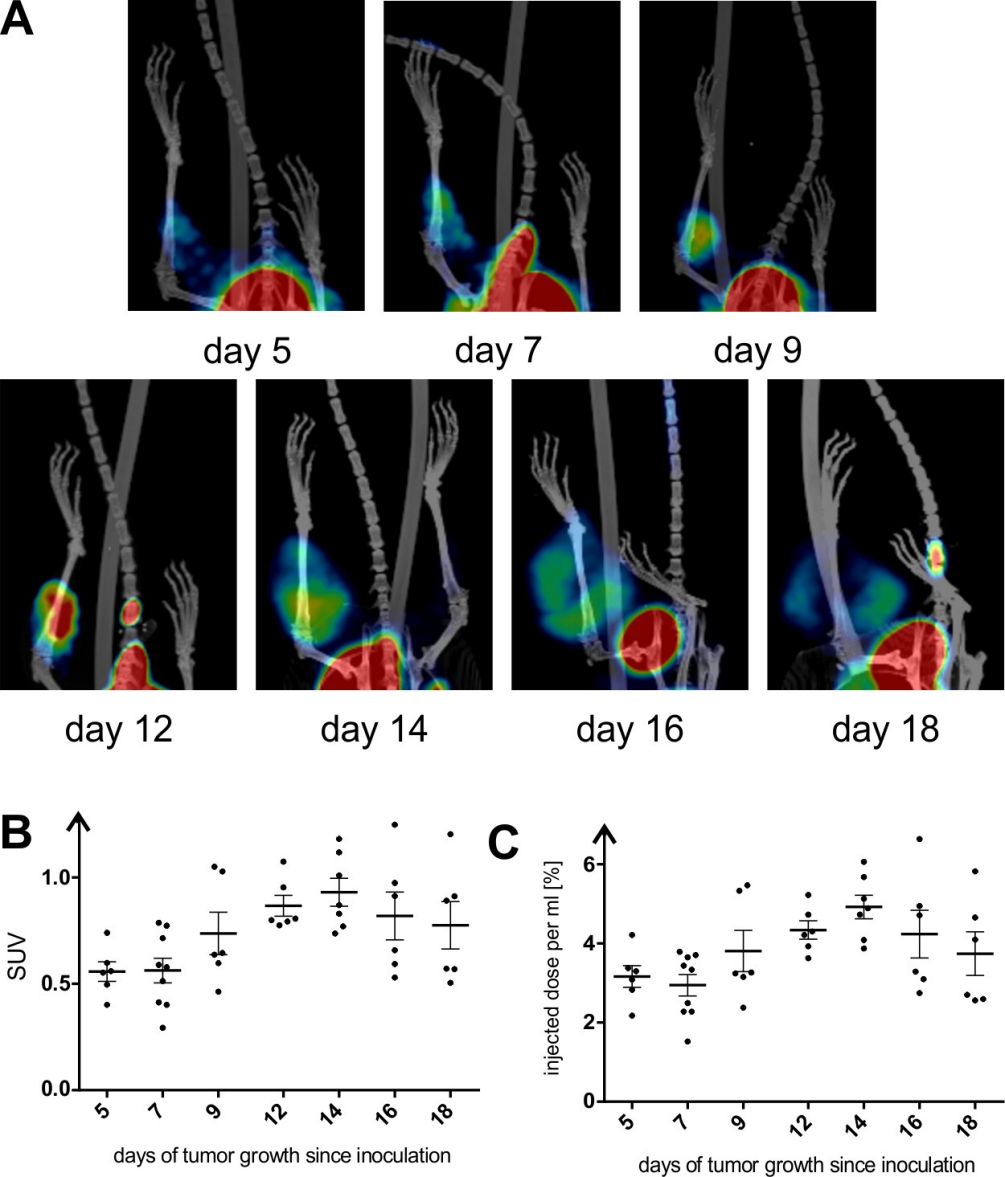
Tumor hypoxia from day 5 till 18 after CT26 cells inoculation in mice measured as a level of [^18^F]FMISO–related radioactivity measured using PET. A–Maximum Intensity Projection (MIP) of fused PET-CT 3D images from day 5 till day 18 of the tumor development. Hot regions indicate the [^18^F]FMISO uptake. B–SUV measurement of the [^18^F]FMISO related signal from the tumor (from day 5 till day 18 of its development) at various stage of development. C–The signal measurement from the tumor (from day 5 till day 18 of its development) reflected as a correctly injected dose per ml of the tissue.

Accumulation of [^18^F]FMISO into hypoxic tissues is commonly accepted. The 3D surface rendered PET-CT image of mouse with 12-day tumor showed high intensity of signal in bladder (due to radiotracer elimination), hypoxic tissue of gastrointestinal track (stomach, intestines, liver) and tumor inoculated on right lower leg (Fig 2). Qualitative analysis of hypoxia extend during tumor development was performed by fusion of PET and CT images of one representative tumor at each time-point (Fig 3A). The degree of tumor hypoxia was dependent on the tumor age. The PET signal gradually enhanced in time during tumor development, reaching the highest readout at the day 14. The 14-day old tumor had a noticeable clear zone (necrosis), surrounded by the high-intensity signal tissue. This zone gradually increased until day 18, when signal-deprived region occupied the majority of the tumor volume (Fig 3A).

Quantitative analysis of hypoxia intensity in different tumors was carried out by counting SUV and %ID/ml for each examined mouse. The SUV analysis (Fig 3B) indicated that hypoxia-related signal subsequently grew since the first time-point (day 5) when it was an average 0.558, till day 14 when it reached an average 0.931. Then, the signal subsequently decreased from day 14. However, the %ID/ml showed that the lowest hypoxia intensity occurred in 7 days old tumors and since this time-point it gradually increased (Fig 3C). Regardless the measurement method, the hypoxia peak was observed at day 14 of tumor development, followed by its gradual decrease.

**Fig 4:**
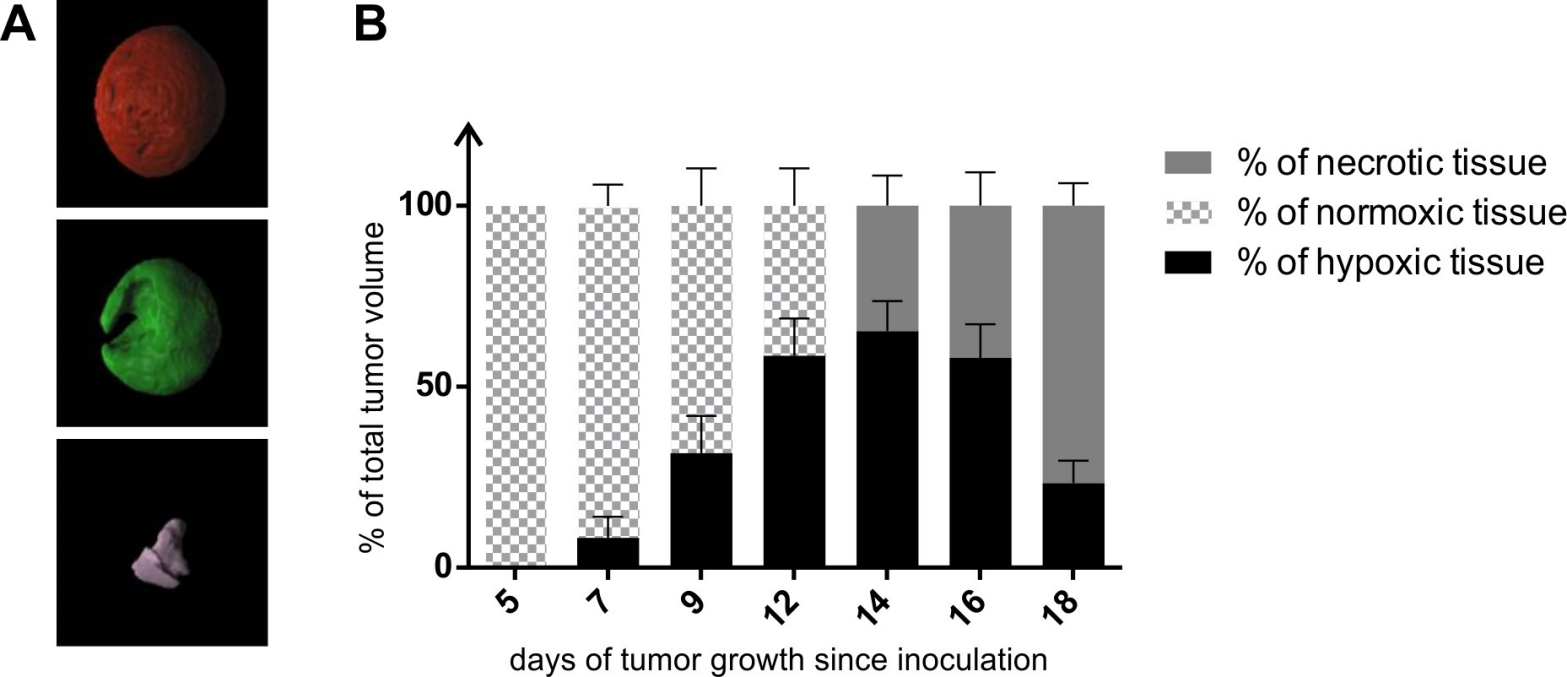
Regions of normoxia, hypoxia and necrosis in developing CT26 tumors. A–Surface rendered tumor (CT26) at the day 16 of development based on the CT measurement (red) and its hypoxic (green) and necrotic (violet) regions based on the [^18^F]FMISO labeling detection using PET. B–Graph showing percentage of CT26 tumor normoxia, hypoxia and necrosis at various stages of its development (from day 5 till day 18) measured using [^18^F]FMISO PET-CT imaging.

Rendered PET signals cover completely CT signals, reflecting tumor shape. Surface rendered CT image of illustrative 16-day old tumor and its rendered PET images of hypoxic and necrotic regions shows Fig 4A. Calculations of the percentage of tumor volume occupied by normoxic tissue, hypoxic tissue and necrotic tissue in relation to total volume of tumor CT image shows that percentage of normoxic tissue gradually decreased till day 14 reaching 0% (Fig 4B). In contrary, percentage of hypoxic tissue gradually increased till day 14 when it started to decrease. Necrotic tissue appeared at day 12 and enlarged subsequently with every time-point occupying 75% of the tumor tissue on day 18 (Fig 4B).

### Microscope analysis

**Fig 5:**
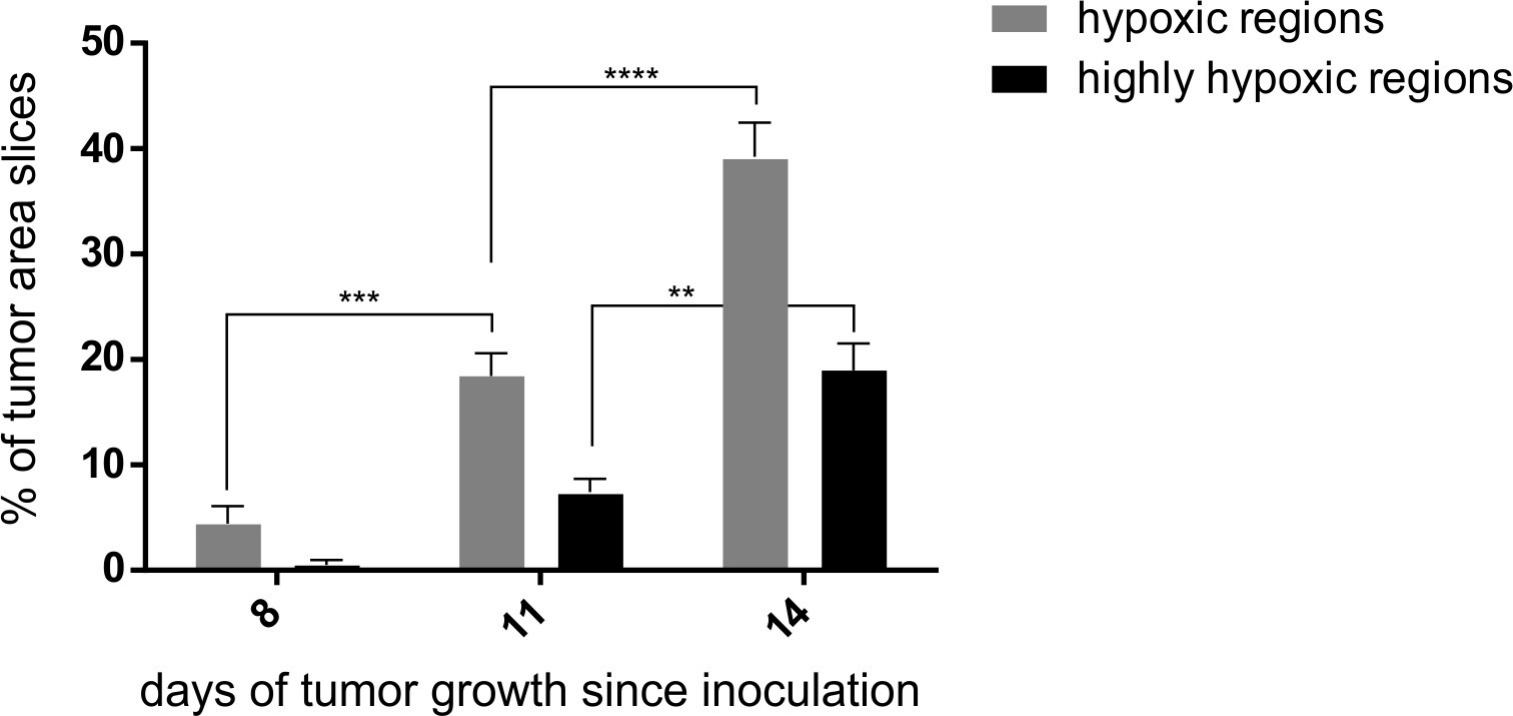
Tumor hypoxia at day 8, 11 and 14 after CT26 cells inoculation in mice measured as a level of PIMO-related fluorescence at fluorescence microscopy; depending on determined threshold of fluorescence the graphs relate to percentage of highly hypoxic regions and percentage of hypoxic regions.

The microscopic analysis of PIMO-labeled regions, reflecting tumor hypoxia, at selected time-points showed that it gradually increased from day 8 till day 14 (Fig 5). This analysis showed that percentage of hypoxic regions at day 8 was only 5, however at day 11 it was almost 20 (p<0.001), reaching 40% at day 14 (p<0.001) (Fig 5). Analysis of the highly hypoxic regions reflected by the highest green fluorescence revealed hypoxia increase from 1% at day 8, to 8% at day 11 (p<0.05) reaching 20% at day 14 (p<0.001) (Fig 5).

## Discussion

Tumor hypoxia is a serious medical problem. Cells become hypoxic when oxygen demand exceeds oxygen supply. Solid tumors are less oxygenated than normal tissues of their origin and the level of their hypoxia affects the curability regardless of treatment methods. That is why the proper analysis of tumor hypoxia at different stages of its development is important, not only for clinicians, but also for scientists in order to better define the biological models. There are various methods of hypoxia evaluation: measurement of partial oxygen pressure with needle electrodes, measurement of hypoxia-induced markers, expression and imaging of tumor microenvironment. Although these methods are used in tumors studies, none of them represents a clear “gold standard”. None of the current methods can completely capture heterogeneities in tumor oxygen levels caused by the complexity of the tumor tissue, complex nature of blood supplies and oxygen consumption [1,3,13]. In this study, we compared endogenous method (gene expression of hypoxia-induced factors) and imaging methods (PET and fluorescence microscopy) in order to determine the location and level of hypoxia at various stages of tumor development. Moreover, we estimated ratio of normoxia/hypoxia/necrosis during the colorectal carcinoma CT26 development in mice.

Low oxygen levels inside the tumor stimulate expression of hypoxia-inducible factor 1α (*HIF1A*) transcription factor [14–17] which regulates expression of more than 100 other genes. One of them is the most potent inducer of blood vessels formation, vascular endothelial growth factor (*VEGFA*) [18–20]. The VEGFA expression level peaks follow the hypoxia level peaks. We interpret, that these reflect neovascularization inside the tumor mass. Our study showed that expression of both mentioned factors (*HIF1A* and *VEGFA*) was the highest et the early stage of tumor development (Fig 1). However, expression of the other hypoxia-specific genes: *ERBB3* and *CA9*, *ANGPTL4* was the highest at later stages of tumor development (Fig 1). These genes are induced by the acidic environment following the previous hypoxic conditions [21–22]. However, this method allows for analysis of the mean gene expression in the whole tumor tissue homogenate. Expression of hypoxia-specific genes can be completely different in various cells and various tissue regions inside the tumor mass.

PET-CT and microscope analysis of the CT26 tumors at various stages of development allowed us to study location, extent and the relative number of hypoxic regions. It was clearly observed that proliferating cells were situated close to a functional blood vessel and necrotic cells were situated in an outer layer. Tissue between the layers of healthy cells and the necrotic cells were chronically hypoxic [4,5]. The PET quantitative analysis using [^18^F]FMISO showed that considering SUV calculations, tumor hypoxia gradually increased since the first time-point (Fig 3B) with subsequent stages of tumor development, reaching the highest value at day 14. Similarly, the %ID/ml value showed that the lowest hypoxia intensity occurred in 7-day old tumors and since this time-point it gradually increased (Fig 3C). Li et al. (2007) showed that pimonidazole positive fraction (PPF) of investigated tumors was the highest at the initial states of growth and gradually decreased until the tumors reached 1–4 mm in diameter. Subsequently, hypoxia in examined samples progressively enlarged up to 10 mm of tumor diameter, which was maximum size of the tumors in that study [23]. Comparing the first time point of [^18^F]FMISO experiment (5 days) with the second one (7 days), decrease of %ID/ml value might indicate the neovascularization of the tumor tissue at the early stage of development. It correlated with qPCR results showing the highest expression of *HIF1A* and *VEGFA* in the first time-points of tumor growth (4 days). Subsequently, both SUV and %ID/ml values showed gradual increase until the tumor age of 14 days. This was a consequence of much faster growth of cancer cells than blood vessels and therefore ineffective oxygen supply. However, decrease in expression of hypoxia-related genes in the subsequent time-points might reflect necrosis of the inner layer of the tumor mass. Small necrotic foci were visible on fused PET-CT images of tumors since day 14 of their development (Fig 3A). The necrotic foci enlarged with every next time-point, occupying most of the tumor volume at the last time-point (Fig 3A). The progression of hypoxia-derived necrosis was the reason of SUV and %ID/ml values decrease at terminal two time-points of tumor growth, due to lack of [^18^F]FMISO distribution to necrotic tissue.

Examination of the [^18^F]FMISO distribution revealed tissue hypoxia at molecular level *in vivo* with high sensitivity and specificity because it reflected dynamic process of tracer uptake over time [24]. In order to additionally confirm these results, we have performed microscopy analysis of PIMO distribution in the tumor tissue. We showed that from day 8 till day 14 it gradually increased (Fig 5). Despite both [^18^F]FMISO and PIMO are labeled nitroimidazoles, PIMO is less accurate because we may observe just randomly selected tissue slides and not the whole tumor tissue in a real time. However, a strong and significant relationship between [^18^F]FMISO-PET and PIMO immunohistochemical results was reported [25–27]. It indicates advantages of [^18^F]FMISO-PET to measure hypoxic sites in whole tumors in a non-invasive manner [25–28]. Furthermore, this method is even more precise than post-mortem microscopic analysis. However, a positive correlation between carbon anhydrates 9 expression and PIMO immunohistochemistry is not a general finding, while the equally significant and strong correlation between *CA9* expression and [^18^F]FMISO autoradiography has been reported [27]. It strengthens the rationale for application of the noninvasive PET method to evaluate tumor hypoxia. Advantages and disadvantages of the all methods used for this study are summarized in Table 1.

**Table 1:** Advantages and disadvantages of three methods used for hypoxia evaluation in solid tumors: Gene expression analysis, PET-CT/18F-FMISO and microscopy/PIMO.

	RT-PCR/Gene expression	PET-CT/^18^F-FMISO	Microscopy/PIMO
**advantages**	1. Examination of the whole organ 2. Examination of changes at molecular level (before the morphologic changes occur)	1. Direct visualization of the whole organ 2. Evaluation of the % of hypoxic regions in the whole tumor	1. Visualization of the whole organ section on the slide 2. Evaluation of the % of hypoxic region in the tumor section
**disadvantages**	1. Results reflect changes in the whole organ without division on the hypoxic, normoxic, necrotic regions 2. The result reflects mean changes in all the regions above	1. Small tumors invisible	1. Results reflect only part of the tumor (just the section) otherwise it is very time-consuming if the whole organ is cut on serial sections

Incorporation of hypoxia information in clinical tumor assessment is important for clinical development of personalized, hypoxia-based therapies, which will ultimately improve outcomes. It is particularly relevant due to recent clinical trial data reports demonstrating importance of tumor hypoxia identification in order to enable appropriate patient classification and to make decisions regarding the therapy management for each patient [6]. Our analysis of the normoxia/hypoxia/necrosis ratio clearly showed that prolonged hypoxia of the tumor tissue leads to necrosis of the inner layer of the tumor mass (Fig 4B). That is why evaluation of tumor hypoxia at various stages of development is important also from the scientific point of view—to better characterize the studied models and understand processes that undergo inside the tumor mass.

Comparison of our results with available studies of tumor hypoxia [23,27,28] shows that during tumor development level of hypoxia fluctuates. There are two peaks of hypoxia. The first one is observed at the very early stage of tumor growth, just before the angiogenic switch (as a result of HIF1α increase and VEGF production). From that moment, along with development of blood vessels, hypoxia gradually decreases. However, at subsequent stage of tumor development, due to faster growth of cancer cells than blood vessels, hypoxia gradually increases because of insufficient blood perfusion. Chronically ischemic, hypoxic tissue transforms into necrotic tissue, following the second peak of hypoxia. Moreover, the results of this paper showed that endogenous methods of hypoxia determination rather reflect changes occurring at the cellular level, however in order to measure and visualize hypoxia in the whole organ or tumor, especially in the later stages of tumor development, PET is the preferred tool.

## Supporting information

S1 Table Sequences of primers used in PCR reactions. (DOCX)

S1 Formulas Equations used in quantitative analyses of PET images: standardized uptake value (SUV) and per cent of injected dose per ml of tumor (%ID/ml). (DOCX)

S1 Dataset Raw data set for Figs 1, 3B, 3C, 4B and 5. (DOCX)
