# Supplementary material for: Changes in hypoxia level of CT26 tumors during various stages of development and comparing different methods of hypoxia determination
Source: PLoS One. 2018 Nov 9;13(11):e0206706. doi: 10.1371/journal.pone.0206706 (PMC6226158; doi:10.1371/journal.pone.0206706)
Supplement: S1 Table — (DOCX) [file pone.0206706.s001.docx]

| **Primer label** | **Gene ID** | **Forward Primer** | **Reverse Primer** | **Amplicon Size** |
| --- | --- | --- | --- | --- |
| *ACTB^*^* | 11461 | GGCTGTATTCCCCTCCATCG | CCAGTTGGTAACAATGCCATGT | 154 |
| *B2M^*^* | 12010 | TTCTGGTGCTTGTCTCACTGA | CAGTATGTTCGGCTTCCCATTC | 104 |
| *GUSB^*^* | 110006 | GGCTGGTGACCTACTGGATTT | GGCACTGGGAACCTGAAGT | 131 |
| *HPRT1^*^* | 15452 | TCAGTCAACGGGGGACATAAA | GGGGCTGTACTGCTTAACCAG | 142 |
| *HSP90AB1^*^* | 15516 | GTCCGCCGTGTGTTCATCAT | GCACTTCTTGACGATGTTCTTGC | 168 |
| *LDHAL6B^*^* | 106557 | GGGCTACAAGCATCTTGAGAG | GACACGTTGCACCTGACTG | 101 |
| *NONO^*^* | 53610 | ACGAACCCTAGCGGAAATTGC | AGGTTGCGGACTGTAAGGGAT | 111 |
| *PPIA^*^* | 268373 | GAGCTGTTTGCAGACAAAGTTC | CCCTGGCACATGAATCCTGG | 125 |
| *RPL13A^*^* | 22121 | AGCCTACCAGAAAGTTTGCTTAC | GCTTCTTCTTCCGATAGTGCATC | 129 |
| *TBP^*^* | 21374 | AGAACAATCCAGACTAGCAGCA | GGGAACTTCACATCACAGCTC | 120 |
| *EEF2^*^* | 13629 | TGTCAGTCATCGCCCATGTG | CATCCTTGCGAGTGTCAGTGA | 123 |
| *VEGFA* | 22339 | GCACATAGAGAGAATGAGCTTCC | CTCCGCTCTGAACAAGGCT | 105 |
| *TGFB1* | 21803 | CTCCCGTGGCTTCTAGTGC | GCCTTAGTTTGGACAGGATCTG | 133 |
| *CCL2* | 20296 | TTAAAAACCTGGATCGGAACCAA | GCATTAGCTTCAGATTTACGGGT | 121 |
| *CCL5* | 20304 | GCTGCTTTGCCTACCTCTCC | TCGAGTGACAAACACGACTGC | 104 |
| *CA9* | 230099 | TGCTCCAAGTGTCTGCTCAG | CAGGTGCATCCTCTTCACTGG | 126 |
| *ERBB3* | 13867 | TCTGCATTAAAGTCATCGAGGAC | CAGCCGTACAATGTGGGCAT | 107 |
| *ANGPTL4* | 57875 | CATCCTGGGACGAGATGAACT | TGACAAGCGTTACCACAGGC | 136 |
| *HIF1A* | 15251 | CCACAGGACAGTACAGGATG | TCAAGTCGTGCTGAATAATACC | 148 |

^*^ tested as possible housekeeping gene
