# Supplementary material for: Changes in hypoxia level of CT26 tumors during various stages of development and comparing different methods of hypoxia determination
Source: PLoS One. 2018 Nov 9;13(11):e0206706. doi: 10.1371/journal.pone.0206706 (PMC6226158; doi:10.1371/journal.pone.0206706)
Supplement: S1 Formulas — (DOCX) [file pone.0206706.s002.docx]

$$SUV=\frac{C_{img}(t)}{\frac{{ID}_{corrected}}{BW-TW}}[\frac{g}{ml}]$$

where:

- *C_img_(t) –* image derived radioactivity concentration, decay corrected to the injection time point [kBq/ml]
- *ID_corrected_ –* injected dose corrected for radioactivity remaining in tail [kBq]
- *BW –* body weight [g]
- *TW –* tail weight [g]

*%ID/ml* $=\frac{C_{img}(t)}{{ID}_{corrected}}\times100 [\frac{\%}{ml}]$

where:

- *C_img_(t) –* image derived radioactivity concentration, decay corrected to the injection time point [kBq/ml]
- *ID_corrected_ –* injected dose corrected for radioactivity remaining in tail [kBq]
