# Supplementary material for: Changes in hypoxia level of CT26 tumors during various stages of development and comparing different methods of hypoxia determination
Source: PLoS One. 2018 Nov 9;13(11):e0206706. doi: 10.1371/journal.pone.0206706 (PMC6226158; doi:10.1371/journal.pone.0206706)
Supplement: S1 Dataset — (DOCX) [file pone.0206706.s003.docx]

**Dataset for figure 1 –** expression analysis (Real-time qPCR) of selected genes related to hypoxia at various stage of tumor development (from day 4 till 19 post CT26 cells inoculation).

| ***ERBB3*** | ***CA9*** | ***CCL2*** | ***VEGFA*** | ***HIF1A*** | ***ANGPTL4*** | ***TGFB1*** | ***CCL5*** |
| --- | --- | --- | --- | --- | --- | --- | --- |
| **Day 4** | | | | | | | |
| 0,112275 | 0,128096 | 0,295567 | 0,161204 | 0,260643 | 0,172414 | 0,173410 | 0,160772 |
| 0,089928 | 0,130152 | 0,226415 | 0,128700 | 0,168729 | 0,136054 | 0,115562 | 0,105300 |
| 0,114416 | 0,180832 | 0,221566 | 0,195313 | 0,254885 | 0,199336 | 0,166205 | 0,150830 |
| **Day 7** | | | | | | | |
| 0,220264 | 0,261552 | 0,203528 | 0,082259 | 0,082259 | 0,154162 | 0,124740 | 0,097498 |
| 0,220264 | 0,261552 | 0,203528 | 0,082259 | 0,082259 | 0,198282 | 0,124740 | 0,097498 |
| 0,216450 | 0,228484 | 0,174216 | 0,073064 | 0,078186 | 0,154162 | 0,112613 | 0,075472 |
| **Day 10** | | | | | | | |
| 0,215672 | 0,240577 | 0,174014 | 0,069332 | 0,069332 | 0,176471 | 0,141176 | 0,084436 |
| 0,193548 | 0,246711 | 0,192802 | 0,079554 | 0,086381 | 0,189994 | 0,126743 | 0,102180 |
| 0,227445 | 0,213068 | 0,178465 | 0,067265 | 0,071361 | 0,190235 | 0,123102 | 0,077081 |
| **Day 14** | | | | | | | |
| 0,230769 | 0,194426 | 0,208914 | 0,118390 | 0,135379 | 0,177883 | 0,130548 | 0,122299 |
| 0,148957 | 0,150527 | 0,123865 | 0,046678 | 0,046678 | 0,132159 | 0,096556 | 0,059230 |
| 0,152284 | 0,145278 | 0,123001 | 0,059207 | 0,066578 | 0,111899 | 0,085421 | 0,063627 |
| **Day 16** | | | | | | | |
| 0,150225 | 0,151057 | 0,127714 | 0,056402 | 0,060386 | 0,124585 | 0,098717 | 0,062048 |
| 0,193050 | 0,213220 | 0,200133 | 0,096339 | 0,102916 | 0,173511 | 0,129702 | 0,098264 |
| 0,145278 | 0,160428 | 0,233827 | 0,148148 | 0,156822 | 0,185874 | 0,167785 | 0,137112 |
| **Day 19** | | | | | | | |
| 0,147348 | 0,164835 | 0,256410 | 0,148957 | 0,158898 | 0,170940 | 0,163845 | 0,140713 |
| 0,203943 | 0,191571 | 0,174723 | 0,071702 | 0,080321 | 0,182260 | 0,142789 | 0,078637 |
| 0,226244 | 0,237154 | 0,180614 | 0,088209 | 0,089874 | 0,187266 | 0,136426 | 0,087133 |

**Dataset for figure 3B** **–** SUV measurement of the [^18^F]FMISO related signal from the tumor

at various stage of development.

| **5 days** | **7 days** | **9 days** | **12 days** | **14 days** | **16 days** | **18 days** |
| --- | --- | --- | --- | --- | --- | --- |
| 0,552810 | 0,513405 | 0,597184 | 0,775147 | 0,736605 | 0,657714 | 0,890805 |
| 0,598607 | 0,412858 | 0,636602 | 0,953607 | 1,118206 | 0,593030 | 0,504458 |
| 0,559782 | 0,293396 | 0,462786 | 0,799601 | 1,009566 | 0,913491 | 0,571374 |
| 0,401071 | 0,787097 | 1,029136 | 0,793023 | 1,181647 | 1,248302 | 0,569538 |
| 0,739733 | 0,714921 | 1,050720 | 1,074490 | 0,829687 | 0,529386 | 1,203619 |
| 0,496028 | 0,589356 | 0,645488 | 0,806315 | 0,870566 | 0,974238 | 0,912248 |
|  | 0,773065 |  |  | 0,770563 |  |  |
|  | 0,401858 |  |  |  |  |  |
|  | 0,579013 |  |  |  |  |  |

**Dataset for figure 3C –** the signal measurement from the tumor (from day 5 till day 18 of its development) reflected as a correctly injected dose per ml of the tissue.

| **5 days** | **7 days** | **9 days** | **12 days** | **14 days** | **16 days** | **18 days** |
| --- | --- | --- | --- | --- | --- | --- |
| 3,296420 | 2,495586 | 3,242438 | 4,213156 | 4,083744 | 3,287420 | 4,097066 |
| 3,373384 | 2,276580 | 3,154228 | 4,727202 | 5,677615 | 4,946626 | 2,561350 |
| 3,086748 | 1,519791 | 2,373260 | 4,302746 | 5,126004 | 3,093773 | 2,700575 |
| 2,176480 | 3,790043 | 5,330929 | 3,929263 | 6,059727 | 4,708110 | 2,596185 |
| 4,215002 | 3,648023 | 5,470364 | 5,222942 | 4,890582 | 6,633731 | 5,823022 |
| 2,826372 | 3,434477 | 3,261274 | 3,627148 | 4,724278 | 2,742224 | 4,654918 |
|  | 3,705079 |  |  | 3,874124 |  |  |
|  | 2,277136 |  |  |  |  |  |
|  | 3,336292 |  |  |  |  |  |

**Dataset for figure 4B –** graph showing percentage of CT26 tumor normoxia, hypoxia and necrosis at various stages of its development (from day 5 till day 18) measured using

[^18^F]FMISO PET-CT imaging.

| **Normoxic regions** | | | | | | |
| --- | --- | --- | --- | --- | --- | --- |
| **5 days** | **7 days** | **9 days** | **12 days** | **14 days** | **16 days** | **18 days** |
| 100,00000 | 100,00000 | 76,84538 | 13,43885 | 0,00000 | 0,00000 | 0,00000 |
| 100,00000 | 100,00000 | 78,04972 | 39,50792 | 0,00000 | 0,00000 | 0,00000 |
| 100,00000 | 100,00000 | 79,97631 | 67,63091 | 0,00000 | 0,00000 | 0,00000 |
| 100,00000 | 100,00000 | 100,00000 | 73,75068 | 0,00000 | 0,00000 | 0,00000 |
| 100,00000 | 100,00000 | 41,76366 | 15,62954 | 0,00000 | 0,00000 | 0,00000 |
| 100,00000 | 100,00000 | 33,65961 | 38,91393 | 0,00000 | 0,00000 | 0,00000 |
|  | 49,87220 |  |  | 0,00000 |  |  |
|  | 76,58205 |  |  |  |  |  |
|  | 99,45455 |  |  |  |  |  |
| **Hypoxic regions** | | | | | | |
| **5 days** | **7 days** | **9 days** | **12 days** | **14 days** | **16 days** | **18 days** |
| 0,00000 | 0,00000 | 23,15462 | 86,56115 | 70,08656 | 61,79464 | 33,980220 |
| 0,00000 | 0,00000 | 21,95028 | 60,49208 | 86,85249 | 26,16954 | 15,859910 |
| 0,00000 | 0,00000 | 20,02369 | 32,36909 | 81,85374 | 89,65651 | 20,957250 |
| 0,00000 | 0,00000 | 0,00000 | 26,24932 | 84,77498 | 32,31953 | 5,685626 |
| 0,00000 | 0,00000 | 58,23634 | 84,37046 | 32,43141 | 82,86501 | 40,143510 |
| 0,00000 | 0,00000 | 66,34039 | 61,08607 | 39,12169 | 43,75066 | 23,85547 |
|  | 50,12780 |  |  | 62,02975 |  |  |
|  | 23,41795 |  |  |  |  |  |
|  | 0,545447 |  |  |  |  |  |
| **Necrotic regions** | | | | | | |
| **5 days** | **7 days** | **9 days** | **12 days** | **14 days** | **16 days** | **18 days** |
| 0,00000 | 0,00000 | 0,00000 | 0,00000 | 29,91344 | 38,20536 | 66,01978 |
| 0,00000 | 0,00000 | 0,00000 | 0,00000 | 13,14751 | 73,83046 | 84,14009 |
| 0,00000 | 0,00000 | 0,00000 | 0,00000 | 18,14626 | 10,34349 | 79,04275 |
| 0,00000 | 0,00000 | 0,00000 | 0,00000 | 15,22502 | 67,68047 | 94,31437 |
| 0,00000 | 0,00000 | 0,00000 | 0,00000 | 67,56859 | 17,13499 | 59,85649 |
| 0,00000 | 0,00000 | 0,00000 | 0,00000 | 60,87831 | 56,24934 | 76,14453 |
|  | 0,00000 |  |  | 37,97025 |  |  |
|  | 0,00000 |  |  |  |  |  |
|  | 0,00000 |  |  |  |  |  |

**Dataset for figure 5 -** tumor hypoxia at day 8, 11 and 14 after CT26 cells inoculation in mice measured as a level of PIMO-related fluorescence at fluorescence microscopy; depending on determined threshold of fluorescence the graphs relate to percentage of highly hypoxic regions and percentage of hypoxic regions.

| **Hypoxic regions** | | | **Highly hypoxic regions** | | |
| --- | --- | --- | --- | --- | --- |
| **8 days** | **11 days** | **14 days** | **8 days** | **11 days** | **14 days** |
| 0,232407 | 26,970330 | 12,086780 | 0,021210 | 14,578020 | 0,908784 |
| 0,161699 | 22,167880 | 12,118840 | 0,020415 | 10,429400 | 1,352221 |
| 0,084053 | 11,001560 | 5,914528 | 0,018298 | 3,778973 | 0,501576 |
| 0,122985 | 12,647990 | 28,257750 | 0,018087 | 4,235443 | 9,729165 |
| 1,092415 | 0,190328 | 32,401110 | 0,099778 | 0,019519 | 5,951092 |
| 0,122135 | 4,119468 | 11,220770 | 0,041820 | 0,233094 | 1,757812 |
| 0,575090 | 0,161384 | 13,034270 | 0,131662 | 0,017212 | 1,357620 |
| 0,933954 | 0,875416 | 21,617260 | 0,156401 | 0,032184 | 3,342454 |
| 1,104232 | 0,268234 | 5,591277 | 0,023040 | 0,021177 | 0,476663 |
| 1,996096 | 4,545056 | 32,175950 | 0,084319 | 1,278509 | 8,261008 |
| 2,335394 | 17,842060 | 56,127320 | 0,092268 | 4,534124 | 35,474930 |
| 5,570767 | 52,217630 | 59,771090 | 0,481655 | 30,888980 | 38,787230 |
| 2,050012 | 18,192450 | 10,924890 | 0,090593 | 7,141294 | 0,787510 |
| 1,202879 | 48,653050 | 61,299410 | 0,119332 | 24,558400 | 28,924090 |
| 0,086379 | 32,289080 | 46,761020 | 0,045819 | 4,337617 | 20,605220 |
| 0,472367 | 0,091105 | 55,469150 | 0,117152 | 0,019257 | 35,607470 |
| 2,426517 | 0,094618 | 51,155880 | 0,042583 | 0,017917 | 21,241780 |
| 4,764019 | 0,165144 | 32,549970 | 0,131327 | 0,035318 | 14,037770 |
| 1,206793 | 7,067883 | 43,075730 | 0,220408 | 0,556891 | 22,050430 |
| 0,072380 | 50,352090 | 56,799090 | 0,022051 | 25,761180 | 26,950570 |
| 8,716356 | 32,458190 | 60,238420 | 0,149473 | 12,177840 | 42,592960 |
| 12,830080 | 7,686418 | 44,345680 | 0,083829 | 2,387723 | 24,527720 |
| 3,025338 | 13,389670 | 55,676580 | 0,037401 | 4,671271 | 34,294130 |
| 0,320945 | 18,101910 | 62,449880 | 0,028284 | 6,972546 | 42,474580 |
| 48,050590 | 16,433830 | 66,848310 | 13,048600 | 2,869332 | 43,604940 |
| 13,017920 | 16,666290 | 71,440740 | 0,251907 | 5,635249 | 46,530870 |
| 5,609483 | 0,056256 | 73,832940 | 0,324979 | 0,028817 | 54,922540 |
| 2,434181 | 10,888350 | 34,894400 | 0,043057 | 0,740357 | 6,242729 |
| 0,092195 | 0,077728 | 52,146660 | 0,014384 | 0,026297 | 27,005800 |
| 12,739830 | 18,430250 | 54,156740 | 0,143976 | 7,045322 | 29,264220 |
|  | 39,597320 | 53,253190 |  | 16,577520 | 16,879470 |
|  | 21,304460 | 15,367010 |  | 9,823795 | 3,137578 |
|  | 39,260150 | 18,924260 |  | 15,155560 | 2,658366 |
|  | 41,547810 | 32,371660 |  | 16,688550 | 13,391560 |
|  | 32,384840 | 31,827140 |  | 17,714520 | 10,904690 |
|  | 24,672990 | 12,745800 |  | 9,131891 | 1,601277 |
|  | 0,098064 | 40,642310 |  | 0,017546 | 9,775585 |
|  | 20,805450 | 69,226650 |  | 4,435965 | 42,490010 |
|  | 40,627300 | 50,154410 |  | 12,479750 | 23,229790 |
|  | 14,860100 | 20,541330 |  | 3,059878 | 5,915642 |
|  | 46,170240 |  |  | 33,700470 |  |
|  | 58,525060 |  |  | 40,230450 |  |
|  | 0,470077 |  |  | 0,021319 |  |
|  | 8,781411 |  |  | 0,373964 |  |
|  | 16,344530 |  |  | 3,029946 |  |
|  | 16,076580 |  |  | 1,289479 |  |
|  | 26,815690 |  |  | 6,868081 |  |
|  | 44,793710 |  |  | 17,448320 |  |
|  | 0,560508 |  |  | 0,018267 |  |
|  | 16,391640 |  |  | 5,138684 |  |
|  | 0,325119 |  |  | 0,018659 |  |
|  | 2,754768 |  |  | 1,147415 |  |
|  | 11,419100 |  |  | 3,445778 |  |
|  | 0,541377 |  |  | 0,023274 |  |
|  | 3,600005 |  |  | 0,115350 |  |
|  | 13,584040 |  |  | 2,317959 |  |
|  | 6,101465 |  |  | 0,850867 |  |
|  | 41,400420 |  |  | 10,945300 |  |
|  | 29,395890 |  |  | 9,778955 |  |
|  | 43,585170 |  |  | 30,312840 |  |
